# New Evidence for P-gp-Mediated Export of Amyloid-β Peptides in Molecular, Blood-Brain Barrier and Neuronal Models

**DOI:** 10.3390/ijms22010246

**Published:** 2020-12-29

**Authors:** Amanda B. Chai, Anika M. S. Hartz, Xuexin Gao, Alryel Yang, Richard Callaghan, Ingrid C. Gelissen

**Affiliations:** 1 School of Pharmacy, Faculty of Medicine and Health, University of Sydney, Sydney, NSW 2006, Australia; acha3237@uni.sydney.edu.au (A.B.C.); zyan8789@sydney.edu.au (A.Y.); 2 Sanders-Brown Center on Aging, University of Kentucky, Lexington, KY 40504, USA; anika.hartz@uky.edu; 3 Department of Pharmacology and Nutritional Sciences, University of Kentucky, Lexington, KY 40504, USA; 4 Research School of Biology and Medical School, Australian National University, Canberra, ACT 2601, Australia; xuexin.gao@anu.edu.au

**Keywords:** P-glycoprotein, ABCB1, amyloid-beta, neuron, SK-N-SH, Alzheimer’s disease

## Abstract

Defective clearance mechanisms lead to the accumulation of amyloid-beta (Aβ) peptides in the Alzheimer’s brain. Though predominantly generated in neurons, little is known about how these hydrophobic, aggregation-prone, and tightly membrane-associated peptides exit into the extracellular space where they deposit and propagate neurotoxicity. The ability for P-glycoprotein (P-gp), an ATP-binding cassette (ABC) transporter, to export Aβ across the blood-brain barrier (BBB) has previously been reported. However, controversies surrounding the P-gp–Aβ interaction persist. Here, molecular data affirm that both Aβ_40_ and Aβ_42_ peptide isoforms directly interact with and are substrates of P-gp. This was reinforced ex vivo by the inhibition of Aβ_42_ transport in brain capillaries from P-gp-knockout mice. Moreover, we explored whether P-gp could exert the same role in neurons. Comparison between non-neuronal CHO-APP and human neuroblastoma SK-N-SH cells revealed that P-gp is expressed and active in both cell types. Inhibiting P-gp activity using verapamil and nicardipine impaired Aβ_40_ and Aβ_42_ secretion from both cell types, as determined by ELISA. Collectively, these findings implicate P-gp in Aβ export from neurons, as well as across the BBB endothelium, and suggest that restoring or enhancing P-gp function could be a viable therapeutic approach for removing excess Aβ out of the brain in Alzheimer’s disease.

## 1. Introduction

The accumulation of amyloid-beta (Aβ) peptides in the brain is a key pathological hallmark of Alzheimer’s disease (AD). These peptides vary between 37–43 amino acids in length and exist in a range of conformations and assembly states, from monomers to oligomers, protofibrils, and finally insoluble fibrils and plaques [1]. Aβ_40_ and the more hydrophobic and aggregation-prone Aβ_42_ are the most common isoforms, with a higher ratio of Aβ_42_: Aβ_40_ being associated with increased neurotoxicity, and accelerated disease pathology and cognitive decline [2]. In the brain, these peptides are constitutively produced in neurons and astrocytes following enzymatic cleavage of the transmembrane amyloid precursor protein (APP), and subsequently rapidly cleared [3,4,5]. Studies in late-onset AD patients have shown that the production rate of Aβ remains unaltered; rather, impaired cellular clearance mechanisms are responsible for their accumulation in the brain [6]. Excess levels of soluble oligomeric Aβ have been demonstrated to impair long-term potentiation, drive synaptic and receptor dysfunction, propagate tau pathology, neuroinflammation, and oxidative stress, and correlate with disease severity [7,8,9].

Both intra- and extracellular Aβ accumulation have been implicated in neurotoxicity, with the former reported to precede the latter [9,10,11,12]. This raises the question of how intraneuronally-generated peptides are able to exit neurons and enter the extracellular space. The hydrophobic, aggregation-prone and highly membrane-associated nature of the Aβ peptide suggests that its constitutive release from cells relies on active transport [13,14]. P-glycoprotein (P-gp), also known as ATP-binding cassette (ABC) transporter B-family subtype 1 (ABCB1) or multi-drug resistance protein 1 (MDR1), is an ATP-dependent exporter protein with broad substrate specificity, that is ubiquitously expressed on cells with barrier or excretory functions [15]. Several studies have provided evidence that P-gp at the blood-brain barrier (BBB) is responsible for Aβ export out of the brain [16]. Consequently, we hypothesised that such a mechanism could also occur in the neuron. However, there remains overall skepticism about the capacity of P-gp to carry these peptides due to their considerably larger size than most known P-gp substrates [14,17].

The aim of the present study was two-fold: firstly, we sought to provide unequivocal evidence that P-gp is able to transport Aβ peptides using in vitro and ex vivo model systems that have been validated previously [18]. Secondly, we investigated the role of neuronal P-gp, in a cell culture system utilised extensively in AD research, to ascertain whether P-gp plays a role at the site of peptide generation and peptide-mediated damage.

## 2. Results

### 2.1. Biochemical Characterisation of the Interaction between Aβ_40_ and Aβ_42_ with P-gp

**Figure 1:**
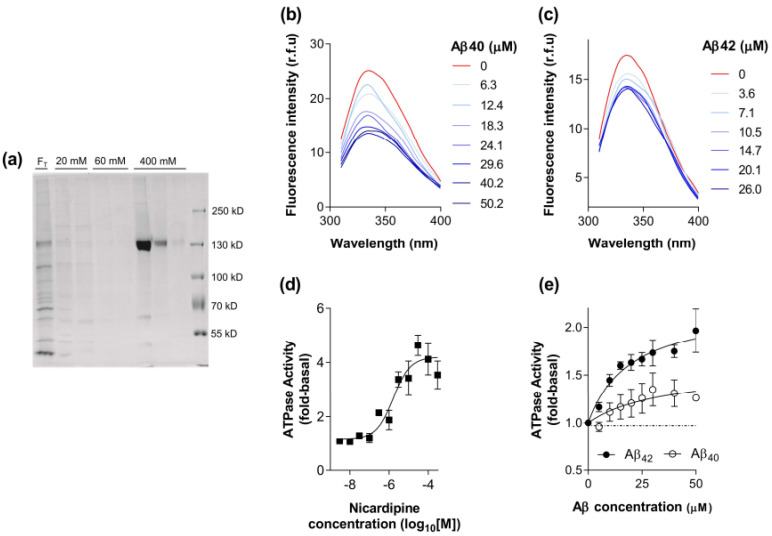
The interaction between Aβ_40_ and Aβ_42_ peptides with purified, reconstituted P-gp. (**a**) Fractions obtained from the metal affinity purification of P-gp were separated on 8% SDS-PAGE and detected with Instant Blue^TM^ protein stain. Fractions (40 μL) from each stage of the purification were loaded onto the gel. F_T_ corresponds to the unbound detergent extracted material, 20 mM and 60 mM represent washing stages, and 400 mM is the elution phase. (**b**) Representative fluorescence emission spectra were obtained for the endogenous tryptophan residues of purified, reconstituted P-gp (15 μg) with excitation at 295 ± 10 nm at 37 °C. Spectra were recorded in the absence or presence of a series of Aβ_40_ concentrations. (**c**) Fluorescence emission spectra obtained as in (**b**), but with varying concentrations of Aβ_42_ peptide. (**d**) ATPase activity of purified, reconstituted P-gp (0.2–0.5 μg) in the presence of varying concentrations of nicardipine. The activity was measured over 40 min at 37 °C and normalised to the value obtained in the absence of drug (basal). Data were fitted by the general dose-response relationship using non-linear least squares regression and values represent mean ± SEM obtained from four independent preparations. (**e**) ATPase activity of purified, reconstituted P-gp (0.2–0.5 μg) in the presence of varying concentrations of Aβ_40_ and Aβ_42_ peptide. The activity was measured over 40 min at 37 °C and normalised to the value obtained in the absence of drug (basal). Data were fitted by a hyperbolic relationship using non-linear least squares regression and values represent mean ± SEM obtained from three independent preparations.

The baculovirus system provides high capacity expression of membrane proteins, including human P-gp with full retention of function including substrate binding [19,20,21]. Figure 1a demonstrates that P-gp was purified to near homogeneity from High-Five membranes and eluted specifically in the presence of 400 mM imidazole. The efficiency of reconstitution was routinely assessed as described previously [21] and the concentration of P-gp obtained in the present investigation was 0.092 ± 0.036 mg/mL (*n* = 4). The total yield was 234 ± 86 μg of purified P-gp per 100 mg of crude High-Five membrane, indicating that the expression was >0.2% of total membrane protein. These parameters are similar to previously published values [21].

The tryptophan quenching assay has been widely used in the field to describe the selectivity and apparent potency of ligand/substrate interaction with P-gp. The affinity is classified as apparent since it measures the binding step and subsequent interaction with a proximal tryptophan residue. Consequently, this assay was used to provide further evidence to support a putative interaction between Aβ peptides and P-gp. A recent publication from our group and collaborators using molecular docking [14] indicated that this interaction is complex, and the kinetics are likely to be slow. Consequently, the assay conditions were configured with an elevated temperature (37 °C) and a longer incubation time than typically adopted for ligand binding studies [22]. As shown in Figure 1b,c, both peptides were able to produce a dose-dependent quenching of the endogenous tryptophan fluorescence intensity, with no shift in the maximal wavelength. The extent of reduction in signal was 20–40% of that produced by the P-gp modulator nicardipine. Numerous researchers [19,23,24,25] have demonstrated considerable variability in the extent of tryptophan quenching by drugs and short peptide substrates of P-gp. These observations suggest that the Aβ peptides have a direct interaction with P-gp that is similar to established drug substrates and inhibitors. This is supported by an earlier study demonstrating that the Aβ peptides quench the fluorescence of an extrinsic probe (MIANS) covalently attached to P-gp [26]. MIANS was attached at the nucleotide-binding domains of P-gp and this indicates a long-distance allosteric interaction, whereas the intrinsic tryptophan quenching is thought to involve residues proximal to the drug binding site and central cavity [23].

Binding to the transporter represents the first step in the process of translocation across the membrane and therefore an activity assay was chosen to further explore the interaction between Aβ peptides and P-gp. Transported substrates of P-gp increase the rate of ATP hydrolysis [27,28,29,30], whereas pure inhibitors reduce the activity [31,32]. Consequently, measuring substrate effects on ATPase activity provides a rigorous assessment of the interaction with P-gp at the level of initial binding and the subsequent coupling event. Figure 1d shows the dose-dependent stimulation of ATPase activity by nicardipine. The overall activity of purified, reconstituted P-gp was characterised with a basal level of 419 ± 30 nmol/min/mg and a maximal activity of 1815 ± 139 nmol/min/mg for the preparations generated (*n* = 4) in this investigation. These values are in agreement with previously published values [21] and represent a 4.3-fold stimulation by nicardipine. Both Aβ peptide isoforms were also able to elicit a stimulation in the ATPase activity of purified, reconstituted P-gp as shown in Figure 1e. The estimated maximal degree of stimulation by Aβ_40_ was 1.5-fold, potentially suggesting that it is a relatively weak substrate of P-gp. Aβ_42_ produced a comparatively greater stimulation of approximately 2.2-fold, which is equivalent, or greater, than established substrates vinblastine, paclitaxel, and rhodamine 123 [19]. It was not possible to generate a more extensive range of concentrations for the peptides (see Methods Section 5.5) and consequently, the affinity cannot be reliably estimated.

Overall, the two assays provide further evidence that Aβ_40_ and Aβ_42_ interact with P-gp and the use of purified, reconstituted protein demonstrates a direct mechanism. In addition, the nature of the effect on ATP hydrolysis may indicate their interaction is akin to a transported substrate, for which there is considerable evidence using cellular systems (for review see [16]).

### 2.2. P-gp Mediates Aβ_42_ Transport from Brain to Capillary Lumen Ex Vivo

Freshly isolated brain capillaries provide a unique ex vivo model of the BBB, which can be used to study endogenous transport processes across the endothelium. P-gp is expressed on the luminal (blood-facing) membrane of the BBB, where it exports substrates from the endothelial cells into the blood [33].

**Figure 2:**
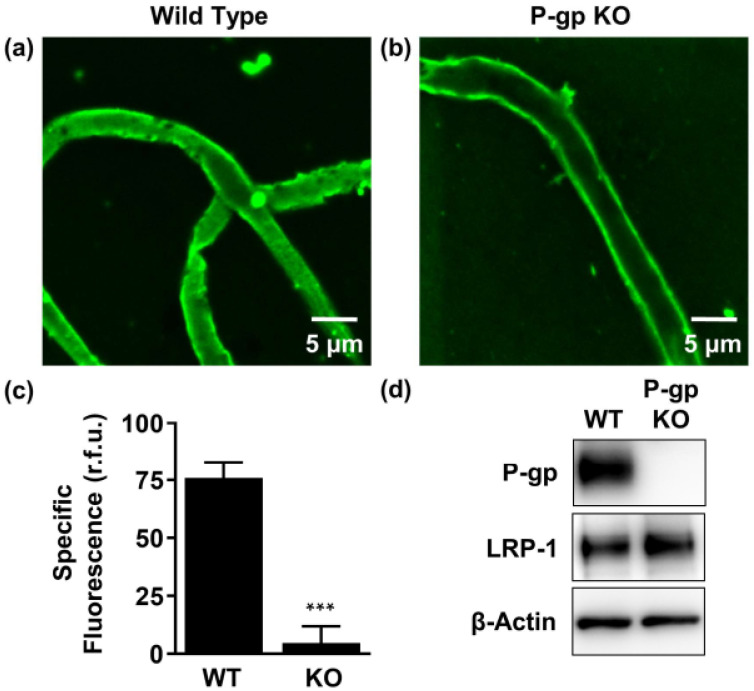
P-gp-mediated human Aβ_42_ (hAβ_42_) transport in isolated brain capillaries. (**a**), (**b**) Representative confocal images showing accumulation of HiLyte^TM^-hAβ_42_ in capillary lumens isolated from wild-type (WT) mice, but not in capillaries from P-gp knockout (KO) mice after a 1-h incubation (steady state; 5 μM HiLyte^TM^-hAβ_42_). (**c**) Data after digital image analysis using ImageJ. Specific fluorescence refers to the difference between total luminal HiLyte^TM^-hAβ_42_ fluorescence and HiLyte^TM^-hAβ_42_ fluorescence in the presence of the P-gp-specific inhibitor PSC833 (5 μM). (**d**) Western blot showing P-gp protein expression in isolated capillaries from WT mice, but not in capillaries isolated from P-gp KO mice. In contrast, LRP-1 is expressed in isolated capillaries from both WT mice and P-gp KO mice. β-actin was used as the loading control. Statistics: data per group are given as mean ± SEM for 10 capillaries from one preparation (pooled tissue: WT (*n* = 10 mice), P-gp KO (*n* = 10 mice)). Shown are relative fluorescence units ((r.f.u.) scale 0–255). *** Significantly lower than control, *p* < 0.001.

To provide a physiological perspective to the binding and transport assays studied in Section 2.1, we compared the accumulation of fluorescent labelled human Aβ_42_ (HiLyte^TM^-hAβ_42_; 5 μM) at steady state in brain capillaries isolated from wild-type (WT) versus P-gp-knockout (KO) mice, using confocal microscopy combined with quantitative image analysis. The Aβ_42_ isoform was selected as it displayed a greater capacity for stimulating ATP-hydrolysis and therefore was deemed a higher affinity substrate of P-gp than Aβ_40_ (Figure 1e). Accumulation of HiLyte^TM^-hAβ_42_ was lower in the lumen of capillaries isolated from P-gp KO mice compared to capillaries isolated from WT mice (Figure 2a,b). Image analysis revealed that luminal HiLyte^TM^-hAβ_42_ fluorescence was significantly (*p* < 0.001) reduced in capillaries from P-gp KO (60.1 ± 4.4 (r.f.u.)) versus WT mice (127.8 ± 5.9 (r.f.u.)), indicating that P-gp is necessary for active Aβ transport from the bath to the vascular space. In concordance, luminal HiLyte^TM^-hAβ_42_ fluorescence was significantly (*p* < 0.001) reduced in WT capillaries treated with the P-gp-specific inhibitor PSC833 (51.9 ± 2.4 (r.f.u.)) versus untreated capillaries (127.8 ± 5.9 (r.f.u.)), whereas fluorescence levels remained comparable between treated (60.1 ± 4.4 (r.f.u.)) versus untreated KO capillaries (55.5 ± 3.9 (r.f.u.)) (Figure 2c). The residual fluorescence present in PSC833-treated capillaries is due to non-specific binding of the HiLyte^TM^-hAβ42 primarily to the endothelial cell surface. Figure 2c shows specific luminal NBD-CSA fluorescence that was taken as the difference between total luminal fluorescence and fluorescence in the presence of PSC833, which represents the P-gp-specific component of HiLyte^TM^-hAβ42 transport. Together, these data indicate that the observed differences in fluorescence accumulation are specific to P-gp-mediated transport. Western immunoblot analysis confirmed high P-gp protein expression in capillary membranes from WT mice and lack of P-gp expression in capillary membranes from P-gp KO mice. In contrast, low-density lipoprotein receptor-related protein 1 (LRP-1), the receptor at the abluminal (brain-facing) membrane of the capillaries responsible for Aβ uptake into the endothelial cells, was detected in capillaries from both WT and P-gp KO mice (Figure 2d).

These results confirm our previous findings showing Aβ transport at the BBB is an active and ATP-dependent two-step process, involving LRP-1-mediated Aβ uptake from the brain into capillary endothelial cells, followed by P-gp-mediated Aβ transport from the endothelium into the capillary lumen [18,34].

### 2.3. P-gp Protein Is Expressed in Human Neuroblastoma Cells

Expression of P-gp protein in neurons has been a point of contention. For example, several groups have demonstrated P-gp expression in neuronally-derived cell lines [35,36,37,38] and on peripheral nerve tissue at the blood-nerve-barrier (BNB) [39,40,41], whereas others have failed to do so or have demonstrated that it is only expressed in the context of brain injury or pathology [42,43,44,45,46,47].

We analysed P-gp protein expression by Western blot in cell lysates obtained from three separate human neuroblastoma cell lines that are regularly used in brain and AD-related research.

**Figure 3:**
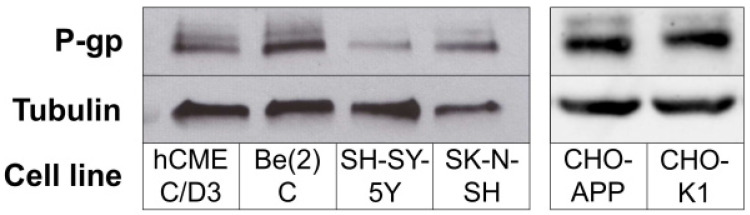
P-gp protein expression in cells. (**Left panel**) Cell lysates obtained from hCMEC/D3 and human neuroblastoma lines Be(2)C, SH-SY-5Y and SK-N-SH were analysed for P-gp protein expression (~170 kDa) via Western blot. (**Right panel**) P-gp expression in the non-neuronal CHO-APP cell line and its parental cell line CHO-K1. Tubulin (~50 kDa) was used as the positive loading control.

Figure 3 (left panel) shows that P-gp could be detected in Be(2)C, SH-SY-5Y and SK-N-SH cells, at levels comparable to those found in human brain endothelial hCMEC/D3 cells, which are commonly used as an in vitro BBB endothelial cell model.

### 2.4. P-gp Is Active and Can Be Chemically Inhibited in CHO-APP and SK-N-SH Cells

Calcein-AM is a soluble hydrophobic non-fluorescent dye that rapidly crosses the plasma membrane and is actively exported by P-gp. When P-gp is active, calcein-AM is efficiently removed from the cell before it can undergo hydrolysis. If P-gp is inactive, intracellular esterases cleave calcein-AM to produce the free acid calcein, which cannot be transported by P-gp and thus remains trapped inside the cell where it produces an intense fluorescence [48]. Therefore, measuring the accumulation of fluorescent calcein is a rapid and sensitive method for studying P-gp activity.

**Figure 4:**
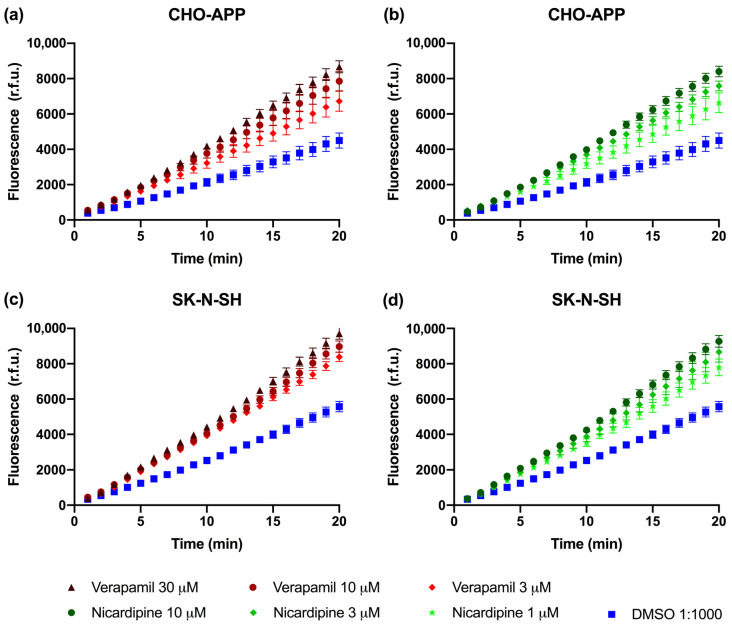
P-gp activity in CHO-APP and SK-N-SH cells. P-gp efflux activity was measured using the fluorogenic P-gp substrate calcein-AM. CHO-APP cells were treated with (**a**) verapamil at 3, 10 or 30 µM or DMSO control, or (**b**) nicardipine at 1, 3, or 10 µM or DMSO control, immediately prior to the addition of 0.1 µM calcein-AM. Similarly, SK-N-SH cells were treated with varying concentrations of either (**c**) verapamil or (**d**) nicardipine or DMSO control. Fluorescence measurements were obtained at 485/535 nm every minute over twenty minutes. Data are presented as the mean ± SEM of three independent experiments, with each condition conducted with six replicates.

Experiments were firstly performed in the CHO-APP cells, which secrete relatively large quantities of Aβ peptides. P-gp protein was confirmed to be expressed in CHO-APP cells at a level consistent with that of the parental CHO-K1 cell line (Figure 3; right panel). Verapamil and nicardipine, both anti-hypertensive calcium channel blockers, were selected due to their well established and strong P-gp inhibitory activity [49,50]. Figure 4a,b show that addition of these P-gp inhibitors to CHO-APP cells increased fluorescence, indicating increased intracellular calcein accumulation, in a concentration-dependent manner. Experiments were subsequently replicated in SK-N-SH neuroblastoma cells, which have previously been established to secrete Aβ_40_ and Aβ_42_ peptides [51,52]. Data show a similar concentration-dependent effect of P-gp inhibition on intracellular fluorescence (Figure 4c,d). Together, these data indicate that P-gp is active and can be chemically inhibited by these two drugs in both cell lines. Furthermore, nicardipine was approximately three-fold more effective than verapamil at inhibiting P-gp-mediated export of calcein-AM (Figure 4), which corresponds with previously published findings [49,50].

Although calcein-AM is a substrate of both P-gp and the related multi-drug resistance transporter ABCC1/MRP1, verapamil and nicardipine do not directly affect ABCC1/MRP1 activity [53,54]. Furthermore, verapamil does not appear to affect the activity of other transporters including ABCG2/BCRP, ABCG4, LRP-1, or RAGE that have been implicated in Aβ transport [55,56,57,58]. Although nicardipine is an effective inhibitor of ABCG2/BCRP [59], expression of this transport protein has not been reported in SK-N-SH and is absent or minimal CHO cells [60,61,62]. Therefore, the observations described in Figure 4 are deemed to be specific to P-gp and not confounded by activity of other ABC transporters.

### 2.5. Cell viability Assays

**Figure 5:**
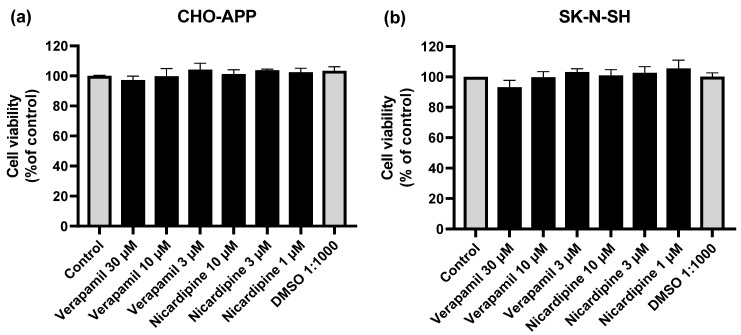
P-gp inhibitors do not affect cell viability. (**a**) CHO-APP and (**b**) SK-N-SH cells were incubated with verapamil or nicardipine at varying concentrations, DMSO, or untreated full culture medium (control) for 24 h to simulate the full experiment duration. The following day, cells were washed and treated with 0.5 mg/mL MTT in serum-free culture medium for two hours at 37 °C. After washing with PBS, cells were lysed with DMSO. Absorbance was measured at 550 nm. Data are presented as mean ± SEM of three independent experiments, each performed with six replicates.

Reduced cell viability compromises the production and secretion of cellular products. Therefore, MTT assays were performed to verify whether incubation with the P-gp inhibitors verapamil and nicardipine at the final experimental concentrations (1–30 µM), would affect CHO-APP or SK-N-SH cell viability. Figure 5a shows that CHO-APP cell viability was not affected by the inhibitors. There was a marginal effect of high verapamil concentrations on SK-N-SH cell viability. However, this was not statistically significant (*p* > 0.05 compared to control) (Figure 5b).

### 2.6. Inhibition of P-gp Reduces Aβ Secretion from CHO-APP and SK-N-SH Cells

**Figure 6:**
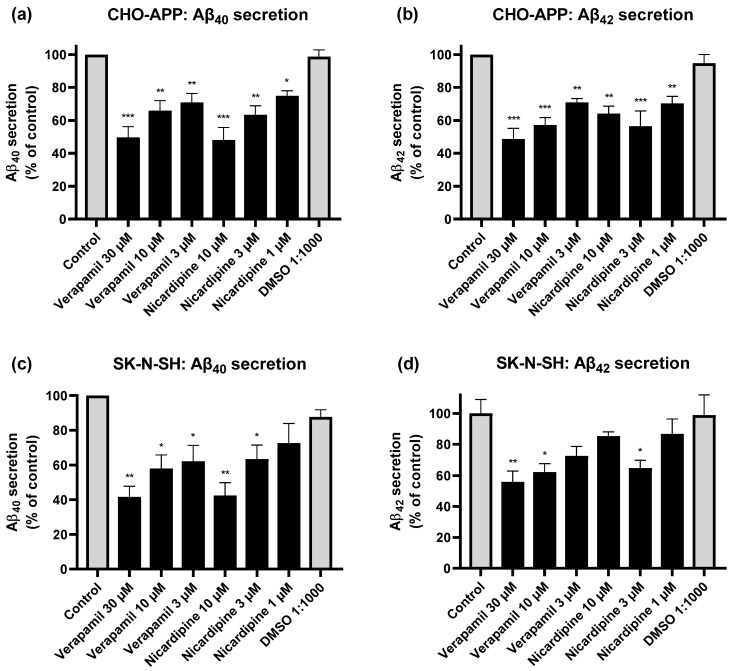
Inhibition of P-gp reduces Aβ efflux from CHO-APP and SK-N-SH cells. CHO-APP cells were treated with verapamil, nicardipine, or DMSO, or left untreated, for 24 h before supernatant was collected and analysed for (**a**) Aβ_40_ and (**b**) Aβ_42_ content by in-house ELISA. Data are displayed as mean ± SEM of three independent experiments for each peptide, and were adjusted for protein concentration. Each experiment was conducted with three biological replicates, each with additional three technical replicates. SK-N-SH cells were similarly treated and analysed for (**c**) Aβ_40_ (mean ± SEM, n = 2) and (**d**) Aβ_42_ (mean ± SEM of one experiment run in triplicate cultures) secretion into media but using commercial ELISA kits. * *p* ≤ 0.05, ** *p* ≤ 0.01, *** *p* ≤ 0.001.

To investigate whether P-gp is involved in the export of Aβ, we assessed the effect of chemical inhibition of P-gp activity on the secretion of Aβ_40_ and Aβ_42_ peptides into cell media. Initial experiments were conducted using CHO-APP cells, which overexpress human APP and exhibit ample P-gp expression. Considering these cells produce large quantities of Aβ, we were able to use in-house ELISAs to quantify these peptides in the supernatant. Control (untreated) cells in our experiments secreted on average 3.3 and 1.1 ng/mL Aβ_40_ and Aβ_42_, respectively. Treatment of CHO-APP with verapamil for 24 h significantly reduced secretion of Aβ_40_ (Figure 6a) and Aβ_42_ (Figure 6b) into the media compared to control in a concentration-dependent manner. Results were most pronounced with verapamil 30 μM, which reduced Aβ_40_ and Aβ_42_ secretion by approximately half, compared to control. Treatment with nicardipine similarly yielded a dose-dependent effect on Aβ_40_ secretion, with 10 μM reducing Aβ_40_ levels to 48 ± 7.6% of that of control (Figure 6a); reductions in Aβ_42_ levels were also significant. However, a dose-dependent relationship could not be confirmed due to variability in response to the highest nicardipine concentration (Figure 6b).

Our observations in CHO-APP are in line with findings from other groups that also report the involvement of P-gp in Aβ export in vitro, utilising human embryonic HEK293 cells transfected with APP695 [26], P-gp-transfected Lewis lung carcinoma cells [63] and LS-180 human colon adenocarcinoma cells [64]. However, the relationship between P-gp and Aβ has, until now, not been investigated in neurons where these peptides are predominantly generated. Therefore, we applied the same experimental conditions to SK-N-SH human neuroblastoma cells.

Since SK-N-SH cells secrete considerably smaller quantities of peptides (approximately 100-fold less than CHO-APP cells), the cell supernatants were analysed using commercial ELISA kits. Control SK-N-SH cells were found to secrete on average 29.0 pg/mL Aβ_40_ and 3.6 pg/mL Aβ_42_. As seen in Figure 6c, pharmacological inhibition of P-gp significantly and dose-dependently reduced Aβ_40_ secretion from SK-N-SH cells. Results were most pronounced with verapamil 30 μM and nicardipine 10 μM, which reduced levels to 42 ± 6.1% and 43 ± 4.0% of that of control, respectively. Verapamil similarly yielded a dose-dependent reduction in Aβ_42_ secretion (Figure 6d). Interestingly, the same observation as seen in CHO-APP (Figure 6b) was also observed in SK-N-SH, with the higher nicardipine concentration producing an unexpected increase in Aβ_42_. Verapamil has been shown not to affect cellular Aβ_40_ or Aβ_42_ production [65], suggesting that the observed reductions in Aβ secretion can be attributable to reduced P-gp-mediated export. As anticipated, SK-N-SH cells consistently secreted higher proportions of Aβ_40_ compared to Aβ_42_ (approximately 8:1 ratio), which corresponds with previously reported in vitro data as well as physiological ratios observed in human AD brains [52,66]. Overall, chemical inhibition using P-gp-specific inhibitors was demonstrated to suppress Aβ peptide secretion from both CHO-APP and SK-N-SH cells.

## 3. Discussion

Positive correlations between P-gp and Aβ transport have previously been described in human and animal studies [16]. However, data from in vitro studies have been more conflicting. The P-gp/Aβ interaction was first proposed by Lam et al., who used a combination of pharmacological inhibition, binding studies, and vesicular transport assays to establish P-gp as an Aβ exporter [26]. Since then, whilst this relationship has been reinforced in several cell models, other groups have reported contradicting data suggesting that P-gp modulation does not affect Aβ transport [16]. In the present study, three lines of evidence collectively point to the involvement of P-gp in the export of Aβ_40_ and Aβ_42_ peptides. Firstly, binding assays utilising purified, reconstituted P-gp demonstrate a direct interaction. Whilst this finding is in line with that reported by Lam et al. [26], the use of intrinsic tryptophan quenching as described here, over quenching of a covalently attached fluorophore probe, has the advantage of stipulating a more direct transporter-peptide binding interaction. Concertedly, Aβ was able to stimulate ATP hydrolysis in a manner comparable with other established P-gp substrates. Secondly, vessels from the P-gp-knockout mice model provided firm physiological evidence for P-gp-mediated transport of the Aβ_42_ peptide at the BBB endothelium. Thirdly, in distinction from previously published in vitro studies that have utilised exogenously applied Aβ peptides [17,26,63,67], our findings from two distinct cell lines (non-neuronal CHO-APP and human neuron-like SK-N-SH) demonstrate that endogenously generated Aβ peptides are transported by endogenously expressed P-gp.

Interestingly, not only do our data indicate that P-gp is expressed and active in neuronal cells, they also show that the extent of inhibition of Aβ efflux by P-gp in SK-N-SH neuroblastoma cells was comparably significant to that in CHO-APP cells. This highlights a previously unappreciated role of P-gp in neurons that not only reshapes our understanding of Aβ pathology, but also has potentially significant implications for drug development and drug-drug interactions. Further studies utilising primary neurons and in vivo models are warranted to confirm the clinical significance of these effects. Notably, although Aβ secretion from both CHO-APP and SK-N-SH cells were significantly reduced in the presence of P-gp inhibitors (Figure 6), secretion was not completely eliminated. This is consistent with previously reported in vitro data [26,63]. This may be attributed to several factors, including those pertaining to the drugs themselves, such as concentration, half-life, and efficacy of inhibition, as well as the involvement of auxiliary peptide export mechanisms such as exosomes and other active transport proteins [55,68,69]. In fact, incomplete elimination of cellular Aβ may be favourable in the context of therapeutic applications [70]. Several reports regard APP and Aβ peptides as serving important physiological roles, including maintaining neuronal function, facilitating brain development, and conferring protection against pathogens [70,71]. Rather, P-gp activity could be a potential target for the development of novel therapeutics in AD to limit the neurotoxic effects of excess Aβ in the brain [64,72,73]. One approach would be to upregulate P-gp function to enhance Aβ export; at the neuron, this could remove intracellularly accumulated peptides, and at the BBB, extracellularly deposited peptides could be cleared. It has been established that intraneuronal Aβ accumulation may be just as toxic, and precedes, extracellular accumulation [10,11]. Hence, alleviating the Aβ load within neurons by facilitating the clearance process out of these cells would be potentially beneficial. However, there is growing evidence that the cell-to-cell spread of misfolded and aggregated proteins, including Aβ peptides, tau proteins and α-synuclein contributes to disease progression in AD as well as other neurodegenerative conditions [74,75,76,77]. In particular, Aβ peptides have been reported to behave as “seeds” that spread in the brain in a prion-like manner [78]. Therefore, further research is necessary to ensure that any transient intermediary extracellular accumulation resulting from increased neuronal secretion of Aβ peptides does not lead to an increase in “seeding” events. In addition, further clarification is required to determine what happens to these peptides once they are exported from their cells of origin. Although not fully elucidated, association with the lipid carrier apolipoprotein E is known to be an important step in the peptide clearance process [79,80]. Critically, any therapeutics aimed at upregulating P-gp function have the potential for drug-drug interactions or off-target effects in patients with comorbidities (such as multi-drug resistant cancers) that must be considered. An alternate recommendation would be to reconsider the prescribing of medications with P-gp-inhibitory effects in patients who are at risk of developing, or have been diagnosed with AD.

As previously mentioned, P-gp expression has been reported in neurons in the periphery at the BNB. It has been suggested that increased permeability and breakdown of the BNB is a contributor to immune- and inflammatory-related neuropathic and neurodegenerative disorders [39,81]. Although expression of P-gp at the BNB has been indicated by several studies to be significantly lower than expression at the BBB [40,41], further studies are needed to determine whether P-gp serves a protective function in these neurons and whether modulation of activity may be beneficial in the prevention of other neurodegenerative disorders.

Lastly, it has been reported that P-gp function declines with ageing, and moreover, Aβ peptides themselves may directly compromise P-gp expression and activity [82,83,84,85]. These factors potentially propagate a vicious cycle that drives AD progression. Therefore, it is imperative to unravel the underlying mechanisms that lead to the decay of P-gp function in the ageing process. So far, post-translational mechanisms such as protein ubiquitination have been described [72,86], which could explain why P-gp expression at the gene level has not yet been identified as a strong genetic risk factor for AD development [87,88]. Further studies are warranted to establish whether curtailing age- and/or disease-related P-gp decline would effectuate any symptomatic improvements or disease-modifying effects.

## 4. Conclusions

The hydrophobic and membrane-anchored nature of the intraneuronally-generated Aβ peptide suggests that simple diffusion, as it has until now been assumed, does not adequately explain the mechanism of its expulsion into the extracellular space. Data presented here provide compelling evidence to substantiate the ability for P-gp to export Aβ. This not only occurs at the BBB endothelium, but for the first time, we have shown that the clearance of Aβ out of neurons is also an active process mediated by P-gp. Further studies are still needed to examine whether modulating P-gp function affects markers of neurodegeneration in vivo, and to confirm if this is a viable avenue to pursue in the search for effective AD therapies. Nonetheless, clarifying the molecular mechanisms involved in the pathway of the Aβ peptide, from its synthesis in the cell to clearance from the brain, is critical for our understanding of the pathophysiology of AD.

## 5. Materials and Methods

### 5.1. Materials

All cell culture materials including media and additives were purchased from Thermo Fisher Scientific (Scoresby, VIC, Australia), except for Hanks’ Balanced Salt Solution (HBSS) which was purchased from Sigma-Aldrich (Castle Hill, NSW, Australia).

For P-gp purification and reconstitution steps (Section 5.3), dodecyl-β-D-maltoside was obtained from Anatrace (Ohio, USA), Ni-NTA His-Bind resin from Merck (Bayswater, VIC, Australia), and SM2 BioBeads from BioRad (Gladesville, NSW, Australia).

Reagents for casting SDS-PAGE gels including Tris-HCl, sodium dodecyl sulfate (SDS) and tetramethylethylenediamine (TEMED) were purchased from VWR Life Science (Tingalpa, QLD, Australia). 40% acrylamide/bis-acrylamide solution was from BioRad (Gladesville, NSW, Australia). Ammonium persulfate was from Sigma-Aldrich. Nitrocellulose membranes and enhanced chemiluminescent reagents were purchased from Merck, GE Healthcare and Pierce (Rockford, IL, USA). The Pierce Bicinchoninic acid (BCA) protein assay kit was purchased from Thermo Fisher Scientific.

(±)-Verapamil hydrochloride and nicardipine hydrochloride were obtained from Sigma-Aldrich. Stock solutions were prepared at 25 μM by dissolving the powders in dimethylsulfoxide (DMSO) and stored at −20 °C. Calcein acetoxymethyl ester (calcein-AM), also obtained from Sigma-Aldrich, was diluted in DMSO to produce a 4 µM stock solution and stored at −20 °C.

Lypholised human Aβ 1-40 (Aβ_40_) and Aβ 1-42 (Aβ_42_) peptides, and HiLyte^TM^-hAβ_42_ were purchased from AnaSpec (Fremont, CA, USA).

Chemicals for brain capillary isolation (Section 5.7) were purchased from Sigma-Aldrich (St Louis, MO, USA). All other reagents, unless otherwise specified, including bovine serum albumin (BSA), IGEPAL, protease inhibitor cocktail, copper (II) sulfate pentahydrate, thiazolyl blue tetrazoliumbromide (MTT) powder, DMSO, glycine, Instant Blue^TM^ protein stain, phosphate buffered saline (PBS), sodium carbonate, sodium bicarbonate, sodium hydroxide, sulfuric acid, and Tetramethylbenzidine (TMB) Liquid Substrate System were obtained from Sigma-Aldrich (Castle Hill, NSW, Australia).

### 5.2. Antibodies

Anti-ABCB1 monoclonal C219 antibodies were obtained from Novus Biologicals and Abcam (Cambridge, MA, USA). Anti-LRP-1 antibody was obtained from Calbiochem-Novabiochem (La Jolla, CA, USA). Anti-β-actin antibody was purchased from Abcam. Anti-α-tubulin monoclonal antibody, and secondary HRP-conjugated anti-mouse and anti-rabbit antibodies were purchased from Sigma-Aldrich. For the in-house Aβ_40_ ELISA, capture and detection antibodies were anti-Aβ_1-40_ (polyclonal rabbit; catalog no. ABN240 from Merck Millipore) and anti-Aβ_1-16_ (monoclonal mouse; clone AB10 from Sigma-Aldrich), respectively. For the in-house Aβ_42_ ELISA, capture and detection antibodies were anti-Aβ_1-16_ and anti-Aβ_37-42_ (polyclonal rabbit; catalog no. Ab34376 from Abcam), respectively.

### 5.3. Purification and Reconstitution of P-gp

A C-terminal dodecyl-histidine version of human P-gp was expressed in *Trichoplusia ni* (High-Five) insect cells using recombinant baculovirus as previously described [19]. Following expression, crude membranes were prepared using differential ultra-centrifugation and stored at −80 °C. P-gp was extracted from the High-Five crude membranes using the detergent dodecyl-β-D-maltoside (DDM) and purified by metal affinity chromatography on Ni-NTA His-Bind resin. Chromatography buffers contained 0.1% (*w*/*v*) DDM and were supplemented with 0.1% (*w*/*v*) of a lipid mixture comprising a 4:1 ratio of *E. coli* total lipid extract and cholesterol. This enabled rapid reconstitution into vesicles by detergent adsorption using SM2 BioBeads.

### 5.4. Tryptophan Fluorescence Quenching Assay for Ligand Binding to Purified, Reconstituted P-gp

Binding of Aβ peptides to purified, reconstituted P-gp was measured by quenching of the intrinsic fluorescence of endogenous tryptophan residues as described [23] and modified [19]. However, imidazole was removed by ultra-centrifugation (70,000 g, 20 min 4 °C) of reconstituted P-gp and subsequent resuspension in binding buffer (20 mM MOPS, pH 8.0, 200 mM NaCl). P-gp (10–15 μg) was added to quartz silica cuvettes in a total volume of 300 μL. A tryptophan fluorescence emission spectrum was measured from 300–400 nm (emission slit width 5 nm) using excitation at 295 ± 10 nm at a scan speed of 120 nm/min. Lyophilised Aβ_40_ and Aβ_42_ peptides were resuspended in 2 mM NaOH at pH~10.5 to concentrations of 1.6 mg/mL (385 mM) and 1.0 mg/mL (221 mM) as described [89]. The peptides were added to sample cuvettes at concentrations in the range of 1–25 μM (Aβ_42_) or 1–50 μM (Aβ_40_). Sample cuvettes were held at a temperature of 37 °C and incubated for 20 min prior to measuring the fluorescence emission spectrum. Nicardipine (5 μM) was added following the final Aβ peptide addition to determine the maximal possible quenching. The spectra obtained in the presence of nicardipine were subtracted from those containing Aβ peptide to remove background signal.

### 5.5. ATP Hydrolysis by Purified, Reconstituted P-gp

The rate of ATP hydrolysis by purified, reconstituted P-gp was determined spectrophotometrically by the liberation of inorganic phosphate as described [19,90]. Samples of P-gp (0.2–0.5 μg) were incubated in 96-well microplates with disodium-ATP (2 mM) and either Aβ peptide (1–50 μM) or nicardipine (10^−9^ − 3 × 10^−4^ M) in a total volume of 50 μL at 37 °C for 40 min. The absorbance (λ = 750 nm) was measured using an iMark plate reader. The activity values were normalised to the basal (i.e., substrate-free) level and plotted as a function of ligand concentration. Data in the presence of nicardipine were analysed using the general dose-response curve:

$$v=v_{initial}+\left(v_{final}-v_{initial}\right)/\left(1+10^{\log\left(EC50-\left[L\right]\right)}\right)$$

as described in [19], where: v is the activity, L is the compound added and EC50 is the potency of effect. Complete dose-response curves were not possible for the Aβ peptides due to poor solubility and their expense from commercial suppliers.

### 5.6. Animals

Animal experiments were approved by the Institutional Animal Care and Use Committee of the University of Kentucky (protocol no.: 2014–1233, PI: Hartz; approved April, 2014) and were carried out in accordance with AAALAC regulations, the US Department of Agriculture Animal Welfare Act, and the Guide for the Care and Use of Laboratory Animals of the NIH.

Male P-gp knockout (KO) mice (CF-1 strain; CF1-Abcb1amds—PGP) and corresponding male CF-1 wild-type (WT) mice were purchased from Charles River Laboratories (Wilmington, MA, USA). Mice were 9 weeks old with an average body weight of 33.7 g (31–35 g) for WT mice and 36.3 g (33–42 g) for P-gp KO mice. All mice were single-housed and kept under controlled environmental conditions (21 °C; 51–62% relative humidity; 12-h light/dark cycle) using an Ecoflo Allentown ventilation system (Allentown Inc., Allentown, NJ, USA). Animals were monitored at least once a day and had free access to tap water and Harlan Teklad Chow 2918 rodent feed (Harlan Laboratories Inc., Indianapolis, IN, USA). After shipping, animals were allowed to acclimate to their new environment for at least 7 days prior to experiments.

### 5.7. Brain Capillary Isolation

Brain capillaries were isolated as previously described [18,72,86]. Mice were euthanised by CO_2_ inhalation and decapitated, brains were removed, dissected, and homogenised in cold PBS buffer (2.7 mM KCl, 1.46 mM KH_2_PO_4_, 136.9 mM NaCl, 8.1 mM Na_2_HPO_4_, 5 mM D-glucose, 1 mM sodium pyruvate, pH 7.4). Ficoll^®^ was added to the brain homogenate to a final concentration of 15% and the Ficoll^®^/brain mixture was centrifuged at 5800× *g* for 15 min at 4 °C. After resuspending the pellet in 1% BSA/PBS, the capillary suspension was passed over a glass bead column to purify capillaries from debris and red blood cells. Capillaries adhering to the glass beads were collected by gentle agitation in 1% BSA/PBS, washed with BSA-free PBS and used for experiments.

### 5.8. Aβ Transport Assay in Isolated Brain Capillaries

To determine P-gp-mediated transport of Aβ, freshly isolated brain capillaries from WT and P-gp KO mice were incubated for 1 h at room temperature with HiLyte™-hAβ_42_ (5 μM; [18,72,86]). For each group, images of 10 capillaries were acquired by confocal microscopy (Zeiss LSM 710 inverted confocal microscope, 40× 1.2 NA water immersion objective, 488 nm line of argon laser, Carl Zeiss Inc., Thornwood, NY, USA). Images were analysed by quantitating luminal HiLyte™-hAβ_42_ fluorescence using ImageJ software v1.48. Specific, luminal HiLyte™-hAβ_42_ fluorescence was taken as the difference between total luminal fluorescence and fluorescence in the presence of the P-gp-specific inhibitor PSC833 (5 μM; [18,72,86]).

### 5.9. Brain Capillary Harvest and Western Blot Analysis

Protein expression levels in brain capillaries were analysed by Western blotting as previously described [18,72,86]. Brain capillaries were homogenised in lysis buffer (Sigma-Aldrich, St Louis, MO, USA) containing Complete^®^ protease inhibitor (Roche, Mannheim, Germany). Homogenised samples were centrifuged at 100,000× *g* for 90 min. Brain capillary membranes were resuspended in buffer containing protease inhibitor and stored at −80 °C.

Western blots were performed using the Invitrogen NuPage^TM^ Bis-Tris electrophoresis and blotting system (Invitrogen, Carlsbad, CA, USA). After electrophoresis and protein transfer, membranes were blocked and incubated overnight with the primary antibody as indicated (P-gp (Abcam): 1:100 (1 μg/mL); β-actin: 1:1000 (1 μg/mL); LRP-1: 1:750 (1 μg/mL)). Membranes were washed and incubated for 1 h with horseradish peroxidase-conjugated ImmunoPure^®^ secondary IgG (1:10,000; Pierce, Rockford, IL, USA). Proteins bands were detected via enhanced chemiluminescence and recorded using a BioRad Gel Doc 2000^TM^ gel documentation system (BioRad, Hercules, CA, USA).

### 5.10. Cell Culture

Chinese hamster ovary cells stably overexpressing human APP (CHO-APP) were a generous gift from Dr. Woojin Kim (Faculty of Medicine and Health, University of Sydney, Sydney, Australia). The cells were generated by transfecting CHO cells with recombinant vectors expressing human 695-amino acid APP cDNA and a puromycin resistance gene, as described [91]. Cells were routinely maintained in F-12 growth medium supplemented with heat-inactivated foetal bovine serum (FBS; 10% *v*/*v*), L-glutamine (2 mM), penicillin (100 units/mL) and streptomycin (100 µg/mL), with the addition of puromycin (7.5 µg/mL), at 37 °C in humidified air containing 5% CO_2_.

SK-N-SH human neuroblastoma cells were obtained from Sigma-Aldrich (Castle Hill, NSW, Australia) and maintained in MEM growth medium supplemented with heat-inactivated FBS (10% *v/v*), L-glutamine (2 mM), penicillin (100 units/mL) and streptomycin (100 µg/mL) at 37 °C in 5% CO_2_.

### 5.11. Cell Harvest and Western Blot Analysis

Cells were washed twice with ice-cold PBS and lysed with cell lysis buffer containing 5 μL/mL protease inhibitor cocktail in IGEPAL. Lysates were syringed with 23-gauge needles to shear cellular DNA and centrifuged at 12,000× *g* rpm at 4 °C for 5 min. Total cell protein concentrations of the resulting supernatants were determined using BCA protein assays. Equal amounts of cell protein were loaded onto 10% (*v*/*v*) acrylamide gels for separation by SDS-PAGE. Proteins were transferred onto nitrocellulose membranes, blocked for 1 h using 5% (*w*/*v*) skim milk or 0.1% (*w*/*v*) BSA in PBS-Tween (0.05% *v*/*v*), then incubated with anti-ABCB1 (1:2000 (Novus Biologicals), overnight 4 °C) or anti-tubulin (1:3000, overnight 4 °C) antibodies. Membranes were rinsed with PBS-Tween, then incubated with HRP-conjugated anti-mouse secondary antibodies (1:10,000) for 1 h at room temperature, and then rinsed again with PBS-Tween. Protein bands were visualised via chemiluminescence and the Bio-Rad ChemiDoc imaging system.

### 5.12. Calcein-AM Assay

P-glycoprotein activity was measured using the calcein-AM assay. CHO-APP and SK-N-SH cells were seeded into 96-well clear-bottom black-walled plates at 4 × 10^4^ cells/well in full culture medium and incubated overnight. The next day when cells reached ~80–90% confluency, media was discarded, and the cells were washed with phenol red-free HBSS or MEM. To assess the inhibitory effect of verapamil and nicardipine on P-gp activity, the inhibitors and DMSO control were prepared at 2× concentrations in HBSS buffer and added to the cells at 100 µL/well. Calcein-AM substrate was also prepared at 2× concentration, and 100 µL was added to each well using a multi-channel pipette to achieve the final working concentrations. Fluorescence measurements were obtained every minute for 20 min, starting immediately after addition of calcein-AM, using the Perkin Elmer Victor X plate reader. The excitation and emission wavelengths were set at 485 and 535 nm, respectively. Temperature was maintained at 37 °C. Measurements were recorded in relative fluorescence units (RFU) and computed using Graphpad Prism software.

### 5.13. MTT Assay

Cells were seeded into a 96-well plate in full culture medium and allowed to adhere overnight. The next day, media was discarded, and cells were incubated with 200 µL/well of verapamil (1–30 µM), nicardipine (1–10 µM), DMSO (1:1000), or no treatment in full culture medium for 24 h. On the day of the experiment, the cells were washed with serum-free medium, to minimise background effect caused by presence of serum. 10X MTT stock (5 mg/mL, prepared in PBS and filter sterilised using a 0.22 µm syringe filter unit) was diluted to 0.5 mg/mL using serum-free medium to yield the working concentration. Using a multi-channel pipette, 100 µL of 1× MTT reagent was added to each well. Cells were incubated for two hours at 37 °C, washed with PBS, then lysed with 100 µL/well of DMSO. The plate was wrapped in foil and placed on a shaker for approximately 10 min to allow even distribution of colour across the wells. Absorbance was measured at 550 nm using a Bio-Rad Microplate Reader.

### 5.14. Preparation of Aβ Peptides

Aβ_40_ and Aβ_42_ solutions were prepared by dissolving lyophilised peptides in 2 mM NaOH (pH~10.5). By avoiding the isoelectric point of Aβ (5.5) in the initial solvation step, aggregation and oligomerisation of the peptide is minimised [89]. For fluorescence quenching assays, Aβ_40_ and Aβ_42_ peptides were prepared fresh at 385 mM and 221 mM, respectively. For ELISA standards, Aβ_40_ and Aβ_42_ 2× solutions were prepared and stored at −80 °C in single-use aliquots. These stock solutions were diluted in PBS (1:1) immediately prior to use to readjust the pH from 10.5 to physiological 7.4.

### 5.15. Enzyme-Linked Immunosorbent Assay (ELISA)

CHO-APP and SK-N-SH cells were seeded onto 24-well plates. The following day, cells were treated with 400 µL/well verapamil, nicardipine, DMSO or plain cell culture media. Because SK-N-SH cells have been reported to secrete low concentrations of Aβ peptides (in the picogram/mL range) [51,52,92,93], an experiment period of 24 h was selected to allow time to accumulate sufficient peptide in the cell supernatant for detection using ELISA. After 24 h, media from each well was collected, treated with 1 µL protease inhibitor cocktail (equivalent to 0.25 mM AEBSF), centrifuged at 3000× *g* RPM at 4 °C for 5 min to remove debris, and used immediately for ELISA (to avoid peptide degradation due to storage and freeze/thawing). Cellular secretion of Aβ_40_ and Aβ_42_ into media was detected and quantified by sandwich ELISA.

For CHO-APP supernatant, a pair of in-house isoform-specific ELISAs were developed: analyses were performed in 96-well Nunc-Immuno^TM^ MaxiSorp plates (Thermo Fisher Scientific) coated with 100 µL per well of capture antibody at 2.5 µg/mL in 0.1 M carbonate buffer (pH 9.5) at 4 °C overnight. Wells were washed four times with PBS-Tween (0.05% *v*/*v*) to remove any unbound antibody, before blocking with 200 µL of blocking buffer (2% BSA, 7.5 g/L glycine in PBS) for 1 h at room temperature. After another four washes, 100 µL peptide standards and samples were loaded into the wells and incubated for 1 h at room temperature on a slow-speed shaker. Washing was repeated, then wells were incubated with 100 µL of the appropriate primary detection antibody (1:1000 in 1% BSA, 3.75 g/L glycine in PBS) for 1 h at room temperature. After washing, 100 µL of secondary horseradish peroxidase-conjugated antibody (1:10,000 in 1% BSA, 3.75 g/L glycine in PBS) was added, and the plate was incubated for 1 h at room temperature. Following another four washes, 100 µL of TMB was added to each well. Plates were incubated in the dark for 30 min at room temperature, then the reaction was terminated by the addition of 50 µL of H_2_SO_4_ (20% *v*/*v*). Absorbance values at 450 nm were measured using a Bio-Rad Microplate Reader. Assay sensitivity was <300 pg/mL for both Aβ_40_ and Aβ_42_.

For SK-N-SH supernatant, the commercial human Aβ_40_ and Aβ_42_ ELISA kits (Invitrogen, catalogue no. KHB3481 and KHB3544) were used in accordance with the manufacturer’s protocols.

Data were computed using Graphpad Prism software; a non-linear 4-parameter regression was used to generate a standard curve, from which the unknown concentrations were determined. ELISA data were adjusted for total protein content of each sample (as determined by BCA protein assays) to account for any potential slight variations in cell viability, by multiplying the ELISA concentration by the ratio of control protein concentration to sample protein concentration.

### 5.16. Statistical Analysis

Data were analysed using Graphpad Prism (v6.01, La Jolla, CA, USA) and Microsoft Excel 2019. Values are expressed as mean ± SEM. MTT and ELISA data were statistically analysed using one-way ANOVA followed by Dunnett’s test. Two-tailed unpaired Student’s *t* test was used to evaluate differences between WT and P-gp KO mice. A *p* value of 0.05 was considered significant (* *p* ≤ 0.05, ** *p* ≤ 0.01, *** *p* ≤ 0.001).

## Data Availability

The data presented in this study are available on request from the corresponding authors.

## Abbreviations

Aβ	Amyloid-beta
ABC	ATP-binding cassette
AD	Alzheimer’s disease
APP	Amyloid precursor protein
ATP	Adenosine triphosphate
BBB	Blood-brain barrier
BNB	Blood-nerve-barrier
Calcein-AM	Calcein-acetoxymethyl ester
CHO	Chinese hamster ovary
ELISA	Enzyme-linked immunosorbent assay
hAβ_42_	Human Aβ_42_
KO	Knockout
LRP-1	Low density lipoprotein receptor-related protein 1
P-gp	P-glycoprotein
WT	Wild-type
